# Supplementary material for: Stem Cell Therapy for Neonatal Hypoxic-Ischemic Encephalopathy: A Systematic Review of Preclinical Studies
Source: Int J Mol Sci. 2021 Mar 19;22(6):3142. doi: 10.3390/ijms22063142 (PMC8003344; doi:10.3390/ijms22063142)
Supplement: Supplementary file 1 [file ijms-22-03142-s001.zip › ijms-1118490-proofed-supplementary/Supplementary Information.docx]

**Supplementary Information**

**Supplementary Table S1** - Studies included in the systematic review and the extracted data from each study: general study design, animal characteristics (animal model, species, sex, age, weight), the protocol for stem cell therapy (source of stem cells, stem cell processing before administration, dose, administration route, timepoint of administration, number of administrations and amount of cells/administration), histological techniques and neurobehavioral tests used, and respective outcomes. (Table in the Excel file "Supplementary Tables 1 and 2")

**Supplementary Table S2** - Stem cell therapy protocols for animal models of hypoxic-ischemic encephalopathy, evaluated by the 58 studies included in the systematic review. (Table in the Excel file "Supplementary Tables 1 and 2")

**Supplementary Table S3** - Motor and cognitive function tests used in the studies included in this systematic review.

| Outcome Evaluated | Behavioral Test | Frequency |
| --- | --- | --- |
| Motor Function | Cylinder rearing test | 19 |
|  | Rotarod | 11 |
|  | Negative geotaxis | 8 |
|  | Open-field paradigm | 5 |
|  | Gait analysis | 3 |
|  | Footprint analysis | 2 |
|  | Hanging wire | 2 |
|  | Adhesive removing test | 1 |
|  | Beam walking test | 1 |
|  | Cliff aversion reflex | 1 |
|  | Elevated body swing test | 1 |
|  | Foot-fault test | 1 |
|  | Forced swimming test | 1 |
|  | Longa Scoring | 1 |
|  | Modified neurological severity scores | 1 |
|  | Righting reflex | 1 |
|  | Vertical pole | 1 |
| Cognitive Function | Morris water maze | 12 |
|  | Novel object recognition test | 5 |
|  | Object in place task | 2 |
| Others | Active Avoidance test | 2 |
|  | Elevated plus-maze | 2 |
|  | Social discrimination test | 1 |

**Supplementary Table S4** - Distribution of the studies using the Rice-Vannucci protocol to induce hypoxic-ischemic lesion and 1.5-2.5 h of hypoxia regarding the animals' age in days.

| Age | Number of Studies |
| --- | --- |
| 7 | 26 |
| 10 | 3 |
| Grand Total | **29** |

**Supplementary Table S5** - Stem cell types used in protocols that used the Rice-Vannucci animal model with 1.5 to 2.5 h of hypoxia in postnatal day seven rats.

| Stem cell type/source | Number of Protocols |
| --- | --- |
| UCB cells | 14 |
| BM-MSCs | 8 |
| UCT-MSCs | 6 |
| UCB-MSCs | 3 |
| PD-MSCs | 2 |
| CD34+ | 1 |
| EPCs/ECFCs | 1 |
| Grand Total | **35** |

**Abbreviations**: BM – bone marrow; EPCs/ECFCs – endothelial progenitor cells/endothelial colony-forming cells; MSC – mesenchymal stem/stromal cells; PD – placenta-derived; UCB – umbilical cord blood; UCT – umbilical cord tissue.

**Supplementary Table S6** - Different routes of administration for stem cell therapy with umbilical cord blood cells in protocols that used the Rice-Vannucci animal model with 1.5 to 2.5 h of hypoxia in postnatal day seven rats.

| Route of Administration | Nb of Protocols |
| --- | --- |
| ICV | 4 |
| IP | 4 |
| IV | 4 |
| IA | 2 |
| Grand Total | **14** |

**Abbreviations**: IA – intraarterial; ICV – intraventricular; IP – intraperitoneal; IV – intravenous.

**Supplementary Table 7** - Different routes of administration for stem cell therapy with mesenchymal stem/stromal cells isolated from the umbilical cord blood & tissue or the placenta, in protocols that used the Rice-Vannucci animal model with 1.5 to 2.5 h of hypoxia in postnatal day seven rats.

| Route of Administration | Nb of Protocols |
| --- | --- |
| ICV | 8 |
| IV | 2 |
| IP | 1 |
| Grand Total | **11** |

**Abbreviations**: ICV – intraventricular; IP – intraperitoneal; IV – intravenous.
